# Conversion of mouse fibroblasts into oligodendrocyte progenitor-like cells through a chemical approach

**DOI:** 10.1093/jmcb/mjy088

**Published:** 2019-01-10

**Authors:** Chang Liu, Xu Hu, Yawen Li, Wenjie Lu, Wenlin Li, Nan Cao, Saiyong Zhu, Jinke Cheng, Sheng Ding, Mingliang Zhang

**Affiliations:** 1 Department of Histoembryology, Genetics and Developmental Biology, Shanghai Jiao Tong University School of Medicine, Shanghai Key Laboratory of Reproductive Medicine, Shanghai, China; 2 Department of Cell Biology, Second Military Medical University, Shanghai, China; 3 Zhongshan School of Medicine, Sun Yat-sen University, Guangzhou, China; 4 The Fifth Affiliated Hospital of Sun Yat-sen University, Zhuhai, China; 5 Life Sciences Institute, Zhejiang University, Hangzhou, China; 6 Department of Biochemistry and Molecular Cell Biology, Shanghai Key Laboratory for Tumor Microenvironment and Inflammation, Key Laboratory of Cell Differentiation and Apoptosis of Chinese Ministry of Education, Shanghai Jiao Tong University School of Medicine, Shanghai, China; 7 Gladstone Institute of Cardiovascular Disease, Department of Pharmaceutical Chemistry, University of California, San Francisco, San Francisco, CA, USA

**Keywords:** small molecules, reprogramming, oligodendrocyte progenitor-like cells, cell fate conversion, demyelinating diseases

## Abstract

Transplantation of oligodendrocyte progenitor cells (OPCs) is a promising way for treating demyelinating diseases. However, generation of scalable and autologous sources of OPCs has proven difficult. We previously established a chemical condition M9 that could specifically initiate neural program in mouse embryonic fibroblasts. Here we found that M9 could induce the formation of colonies that undergo mesenchymal-to-epithelial transition at the early stage of reprogramming. These colonies may represent unstable and neural lineage-restricted intermediates that have not established a neural stem cell identity. By modulating the culture signaling recapitulating the principle of OPC development, these intermediate cells could be reprogrammed towards OPC fate. The chemical-induced OPC-like cells (ciOPLCs) resemble primary neural stem cell-derived OPCs in terms of their morphology, gene expression, and the ability of self-renewal. Upon differentiation, ciOPLCs could produce functional oligodendrocytes and myelinate the neuron axons *in vitro*, validating their OPC identity molecularly and functionally. Therefore, our study provides a non-integrating approach to OPC reprogramming that may ultimately provide an avenue to patient-specific cell-based or *in situ* regenerative therapy.

## Introduction

Demyelinating diseases, such as multiple sclerosis, are among the most disabling and costly neurological disorders, which affect millions of people worldwide. Demyelinating diseases are characterized by the loss or dysfunction of myelin, a process called demyelination. Demyelination impairs saltatory nerve conduction, leads to motor and cognitive deficits, and is considered as one of the major causes of neurological mortality and morbidity (Franklin and Ffrench-Constant, 2008; Fancy et al., 2011). In central nervous system (CNS), myelin is produced by oligodendrocytes (OLs), and is able to ensheath axons during brain development and remyelinate axons after brain damage (Sherman and Brophy, 2005). OLs are differentiated from oligodendrocyte progenitor cells (OPCs), the resident progenitors in the CNS during development and adulthood (Dawson et al., 2003). Notably, in animal transplantation studies, it is the OPCs, the lineage-restricted precursor population, rather than neural stem cells (NSCs) or mature OLs, survived and readily produced OLs and subsequently myelinated axons of neuron *in vivo*, supporting that they could be an ideal cell population for clinically treating demyelinating diseases (Franklin and Ffrench-Constant, 2008; Goldman et al., 2012). Therefore, it has sparked the interest in treating demyelination diseases by enhancing the generation of OPCs.

To obtain functional OPCs, it has been reported that OPCs differentiated from pluripotent stem cells (PSCs, including embryonic stem cells, ESCs, and induced pluripotent stem cells, iPSCs) (Brustle et al., 1999; Goldman et al., 2012; Wang et al., 2013), or those reprogrammed from fibroblasts via ectopically expressing a defined set of transcription factors (TFs) (Najm et al., 2013; Yang et al., 2013), remyelinate axons in an animal model of myelin disease. However, to apply a similar and effective approach to human disease, we must overcome the limited access to patient-specific autologous sources of PSCs, or potentially harmful effects caused by integration of exogenous transgenes during TF-mediated reprogramming, respectively. Alternatively, chemical-induced cellular reprogramming has been established and provides a non-integrating strategy to generating desired cell types. Recently, chemical-based reprogramming for cell types, such as iPSCs (Hou et al., 2013; Zhao et al., 2015; Cao et al., 2018), NSCs (Cheng et al., 2014; Zhang et al., 2016), neurons (Hu et al., 2015; Li et al., 2015; Zhang et al., 2015), cardiomyocytes (Fu et al., 2015; Cao et al., 2016), and multipotent progenitor cell types (Wang et al., 2016; Han et al., 2017), have been established. However, the transgene-free approach to generation of OPC, the lineage-restricted precursor population, which is the most relevant cell type to clinically treating demyelinating diseases through cell-based therapy, has not yet been reported, and is thus highly desired.

During reprogramming to iPSCs, cells induced by both TFs and chemicals go through mesenchymal-to-epithelium transition (MET) (Takahashi and Yamanaka, 2006; Zhao et al., 2015). MET process represents an essential stage for successful cell fate conversion (Li et al., 2010), and those MET intermediate cells generated at the early phase of reprogramming bridge starting fibroblasts to target cell types (Zhao et al., 2015). Interestingly, those METed cells are plastic, and the cell fate of those cells could be redirected towards other lineages according to the culture condition, a paradigm that was described as cell-activation and signaling-directed (CASD) reprogramming (Zhang et al., 2014). As a support, multiple cells types, such as neural cells and hepatocytes, were obtained through a transient overexpression of Yamanaka factors (Kim et al., 2011; Zhu et al., 2013, 2014), or pre-treating cells with iPSC-producing chemical cocktails (Li et al., 2017). However, whether a reprogramming chemical cocktail, which is originally developed to induce trans-differentiation towards cell types other than iPSCs, could activate and subsequently redirect cells towards alternative lineages is still unknown.

We recently established a chemical strategy for NSC conversion (Zhang et al., 2016). In brief, the chemical condition M9 specifically induced the expression of neural genes in mouse embryonic fibroblasts (MEFs), and promoted MET. We therefore attempted to direct the METed cells towards OPCs without first going through established NSC fate. Here, we report that MEFs could be induced to OPC-like cells (ciOPLCs) through chemical strategy. The resultant ciOPLCs resemble primary NSC-derived OPCs molecularly and functionally, and could produce mature OLs that possess the ability of myelination *in vitro*, implying their clinical potentials. Our study provides an alternative way to generate OPCs through chemical-induced reprogramming, which may serve as a potential therapeutic strategy for treating demyelinating diseases.

## Results

### M9 specifically initiates neural program in MEFs

**Figure 1:**
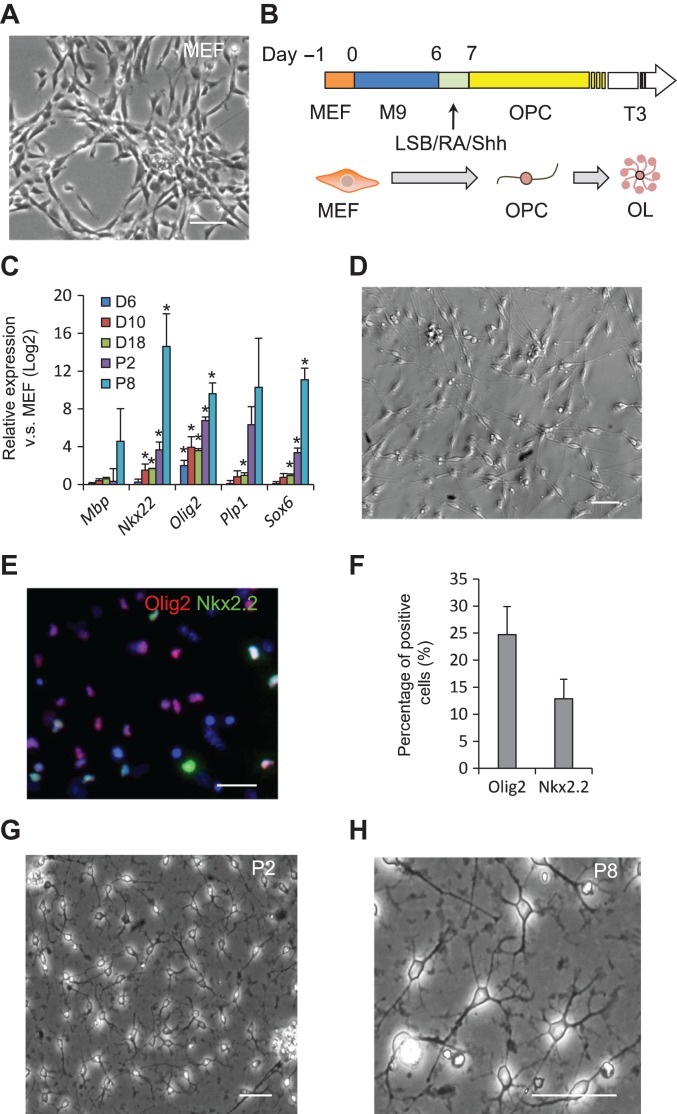
Reprogramming of MEFs into ciOPLCs. (**A**, **D**, **G,** and **H**) Morphology of starting MEFs (**A**), bipolar cells on Day 14 of reprogramming (**D**), ciOPLCs at passage 2 (**G**), and passage 8 (**H**), respectively, was shown. All scale bar, 50 μm. (**B**) Schematic diagram illustrating the protocol for chemical-induced fibroblasts-to-ciOPLCs reprogramming. LSB, LDN193189, and SB431542. (**C**) qRT-PCR analysis showing the expression of indicated genes for samples at indicated time points. Relative expression (Log2) was normalized to MEFs. Data are represented as mean ± SEM. *P < 0.01. (**E** and **F**) Immunostaining analysis of Olig2^+^ and Nkx2.2^+^ cells by Day 14 (**E**), and the percentage of positive cells was analyzed by InCell software from three independent experiments. Data are represented as mean ± SEM.

Our recent study established a chemical condition M9, containing CHIR99021, LDN193189, A83-01, Hh-Ag1.5, retinoic acid (RA), SMER28, RG108, Parnate, and basic fibroblast growth factor (bFGF), that enables neural reprogramming in MEFs (Zhang et al., 2016). During neural reprogramming, M9 specifically induces neural gene expression, and promotes MET, a process that is also observed in paradigms of TF- and chemical-induced PSC reprogramming (Takahashi and Yamanaka, 2006; Li et al., 2010; Zhang et al., 2014; Zhao et al., 2015). Interestingly, the MET process would produce essential and plastic reprogramming intermediates (i.e. METed cells), from which, most, if not all of the resultant target cells, such as iPSCs or chemical-induced NSC-like cells (ciNSLCs), were derived (Li et al., 2010; Zhao et al., 2015; Zhang et al., 2014, 2016). With this in mind, we took the advantage of the neural induction activity of M9, and hypothesized that the cell fate of METed intermediates could be further specified towards OPC through modulating the culture condition. To this end, MEFs, with neither detectable expression of NSC or OPC genes (Supplementary Figure S1A–D), nor differentiation potentials towards neurons or OLs (Supplementary Figure S1E), were seeded into Matrigel-coated 24-well plate as 10000 per well for chemical reprogramming (Figure 1A and B). After an overnight culture, M9 condition was applied, and cells were cultured at 37°C in 5% O_2_ and 5% CO_2_ (Figure 1B). Consistent with previous observation, colonies that underwent MET were emerged towards Day 6 (Supplementary Figure S2B). Quantitative RT-PCR (qRT-PCR) results revealed a rapid induction of neural genes in response to the chemical treatment, although the expression level was much lower at this stage than that in primary NSCs or NSC line (Supplementary Figure S3A–E). Indeed, unlike the primary NSCs, these METed cells on Day 6 failed to generate neurons or OLs when isolated and cultured for 10 days under differentiation conditions, suggesting that they had not established NSC identity at this time (Supplementary Figure S4A–C). Notably, the expression of genes for other lineages in METed cells, including those for pluripotency, endoderm, and mesoderm development, was low and comparable to that in MEFs, suggesting a specific induction toward neural lineage (Supplementary Figure S5A–D). This early-stage neural induction was validated by RNA-seq results (Supplementary Figures S3F and S5E). Interestingly, the generation and maintenance of METed cells are M9-dependent. When M9 was withdrawn from Day 6 onwards, those METed colonies regressed gradually (Supplementary Figure S2A and B), and the expression of neural genes decreased accordingly (Supplementary Figure S3A–E), indicating that those METed cells on Day 6 may represent an unstable reprogramming intermediate.

### Reprogramming of METed cells towards OPC fate

The METed cells generated during reprogramming were reported plastic to cell fate conversion (Kim et al., 2011; Zhang et al., 2014; Zhu et al., 2014; Li et al., 2017). We therefore attempted to induce these uncommitted METed cells towards OPC fate by rationally modulating key signaling pathways that orchestrate OPC development. To this end, we treated METed cells on Day 6 with medium containing LDN193189 and SB431542 (LSB), dual SMAD inhibitors that induce PSCs to neural fate (Chambers et al., 2009), and RA and sonic hedgehog (SHH), which are reported to be able to pattern NSCs to dorsal-ventral OPC fate (Noll and Miller, 1993) for one day (Figure 1B). This treatment led to the upregulation of OPC-related master TFs, such as oligodendrocyte transcription factor 2 (Olig2) and Nk2 homeobox 2 (Nkx2.2) (Figure 1C). We then cultured the cells in the OPC medium containing bFGF, platelet-derived growth factor-AA (PDGF-AA), and SHH afterwards, which favors the specification and maintenance of OPCs *in vitro* (Figure 1B) (Najm et al., 2013). By Day 14, cells with bipolar morphology that resemble OPC were evident (Figure 1D and Supplementary Figure S6A). These changes in gene expression and morphology indicate that these METed cells could be directed towards OPC fate. Consistently, typical OPC genes, such as Nkx2.2, Olig2, and proteolipid protein 1 (Plp1), were gradually induced as detected by qRT-PCR (Figure 1C). Immunostaining showed that ~24.72% and 12.88% of cells were stained positive for Olig2 and Nkx2.2, respectively (Figure 1E and F). No Olig2-positive bipolar cell was observed in control conditions without M9 treatment for the first 6 days, or without OPC specification from Day 6 afterwards (Supplementary Figure S6B and C), suggesting that the initial neural induction activity of M9 and subsequent specification are both required for OPC reprogramming. Those cells were then trypsinized and subjected to suspension culture in OPC medium, which, within ~3–5 days, formed floating spheres (passage 0, P0). The spheres were transferred into Matrigel-coated plates, and after propagation grew as a homogeneous population with typical bipolar or tripolar morphology (Figure 1G), which could be maintained in the OPC medium for at least eight passages (Figure 1H). We hereby referred to those cells as ciOPLCs.

### Characterization of ciOPLCs

**Figure 2:**
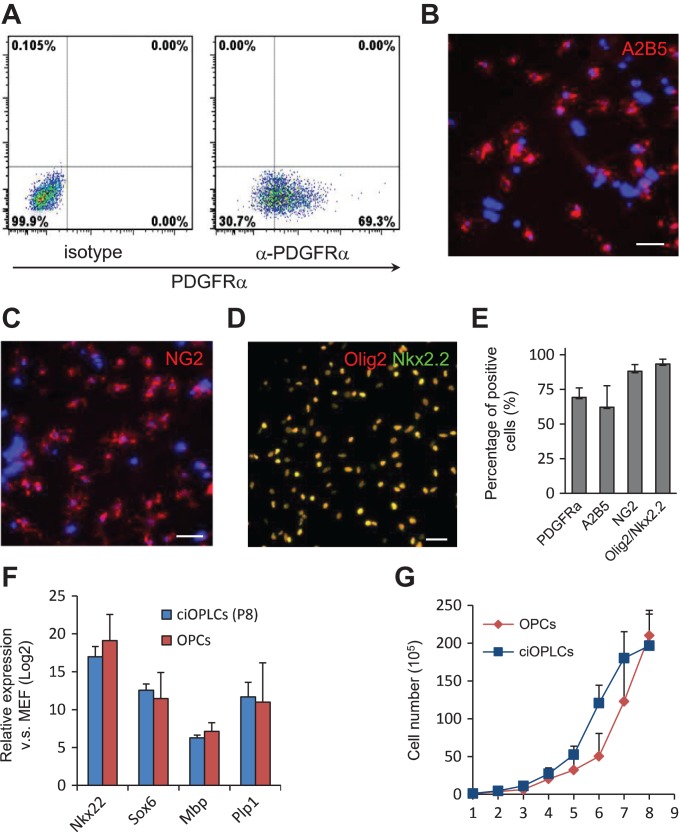
Characterization of ciOPLCs. (**A–E**) Analysis of indicated markers for ciOPLCs by FACS (**A**) and immunostaining analysis (**B–D**), and the quantification (**E**). All scale bar, 50 μm. (**F**) qRT-PCR analysis showing the expression of indicated genes for ciOPLCs at passage 8 (ciOPLCs P8) and primary NSC-derived OPC (OPC). Relative expression (Log2) was normalized to MEFs. (**G**) Growth curve showing the cell number of OPCs and ciOPLCs over passages 1–8. A total of 1 × 10^5^ cells were used for initial experiment. Data are represented as mean ± SEM.

To validate the OPC identity, we characterized these ciOPLCs. Flow cytometry analysis showed that ~69.3% of ciOPLCs at passage 1 expressed PDGF receptor α (PDGFRα, Figure 2A and E), a cell surface receptor highly enriched in OPCs in the developing spinal cord and brain (Pringle et al., 1992). Consistently, other OPC-specific surface markers, including A2B5 (~62.2%), and neural/glial antigen 2 (NG2, ~88.1%), and OPC master TFs, Olig2, and Nkx2.2 (~98.4%), were also detected by immunostaining (Figure 2B–E). QRT-PCR revealed that the expression level of these OPC genes in ciOPLCs was similar to those in primary NSC-derived OPCs (OPCs, Figure 2F). When serially passaged in the OPC medium, ciOPLCs displayed a comparable proliferation rate with OPCs (Figure 2G). These lines of evidence collectively support the conclusion that ciOPLCs have an OPC identity.

### ciOPLCs are able to generate mature oligodendrocytes

**Figure 3:**
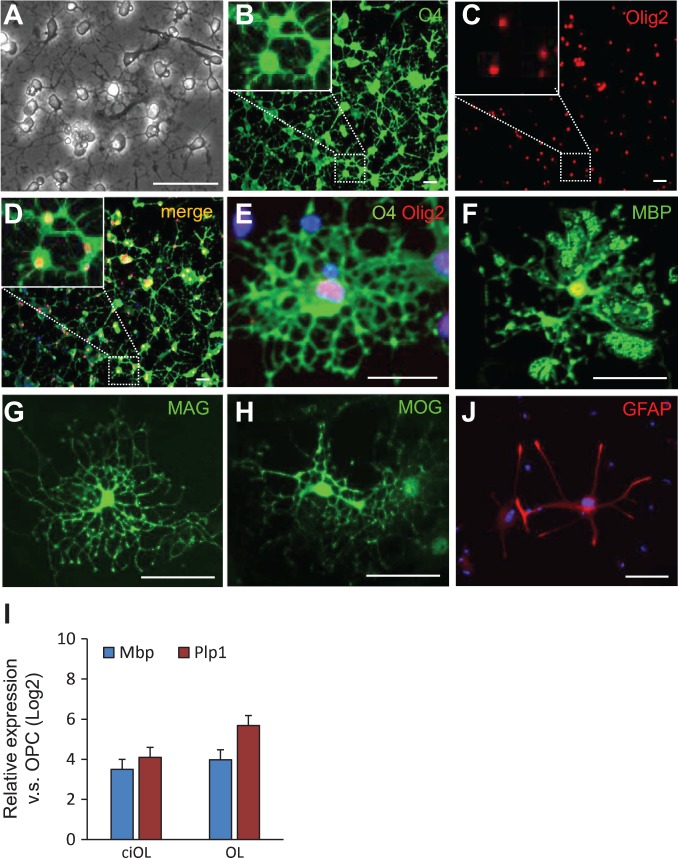
Characterization of the differentiation potential of ciOPLCs. (**A**) Morphology of OL-like cells derived from ciOPLCs. (**B–J**) Immunostaining analysis showing that ciOPLCs can differentiate into OLs (**B–H**) and astrocytes (**J**) that are positive for indicated markers. The enlarged view of the boxed area was shown as insets in **B–D**. All scale bar, 50 μm. (**I**) qRT-PCR analysis showing the expression of indicated genes for OLs derived from ciOPLCs (ciOL) or from primary NSCs (OL). Relative expression (Log2) was normalized to OPCs. Data are represented as mean ± SEM.

To evaluate the differentiation potential, we induced ciOPLC differentiation. Upon differentiation, ciOPLCs stopped proliferating and the cell morphology dramatically changed within 3–5 days. OL-like cells with multiple branches were observed after differentiation for 8–10 days (Figure 3A). By Day 10 after differentiation, O4-positive cells with OL-morphology were readily detected by immunostaining, which co-express Olig2 (Figure 3B–E). Importantly, most of those cells expressed myelin basic protein (MBP, Figure 3F), as well as other defining myelin-related mature oligodendrocyte markers, including myelin-associated glycoprotein (MAG), and myelin oligodendrocyte glycoprotein (MOG) (Figure 3G and H). The expression of OL-specific genes in differentiated cells (ciOL) was comparable to primary NSC-derived OLs (OL, Figure 3I), collectively demonstrating that the ciOPLCs are able to generate mature OLs *in vitro*. In addition, a few glial fibrillary acidic protein (GFAP)-positive process-bearing astrocytes were also detected when ciOPLCs were treated with 10% FBS for 8–10 days (~1.12%, Figure 3J), while we did not find microtubule-associated protein 2 (Map2)-positive neurons under the neuron differentiation condition (data not shown). These results are consistent with previous reports that OPCs are bipotent, and able to produce OLs and astrocytes *in vitro* (Kondo and Raff, 2000; Wang et al., 2013; Yang et al., 2013), and further support a specified gliogenic, but not neurogenic potential, of ciOPLCs.

### ciOPLCs could differentiate into myelinating oligodendrocytes

**Figure 4:**
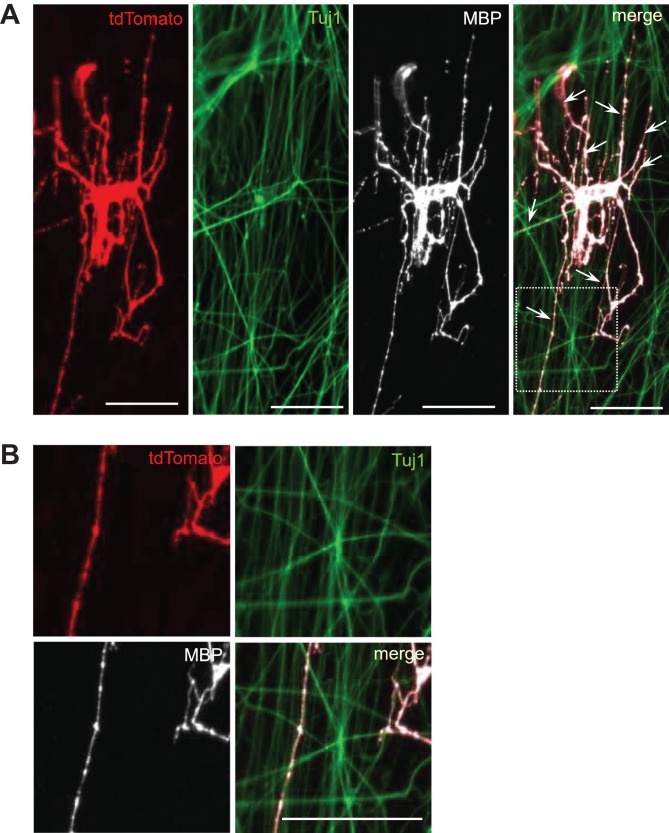
*In vitro* myelination assay validating the functionality of ciOPLCs. (**A** and **B**) Immunostaining analysis showing that td-ciOPLCs can differentiate into td-OLs (tdTomato-positive) that express MBP, and myelinate the axons (Tuj1-positive) *in vitro*. The zoomed images for individual channels and merged image in the boxed area in **A** are shown in **B**. Arrows indicate the myelin. Scale bar, 50 μm.

OPC has the ability to produce mature OLs and subsequently myelinate axons. To further evaluate their myelinogenic potential, ciOPLCs were tested *in vitro* through a co-culture assay (Najm et al., 2013; Yang et al., 2013; Zhang et al., 2016). To distinguish and validate the fibroblasts-originated ciOPLCs, we used tdMEFs for ciOPLC reprogramming (td-ciOPLCs) and the subsequent co-culture assay. As previously described, tdMEFs, which are genetically traced via validated fibroblast specific protein 1 (Fsp1)-Cre/Rosa26-tdTomato system, are permanently labeled with tdTomato expression and can be used for lineage-tracing of fibroblasts and their derivatives. To perform the *in vitro* myelination assay, purified rat dorsal root ganglion neurons (DRGs) were seeded as dense beds of axons for one week, and the td-ciOPLCs derived from tdMEFs were then seeded onto those pre-established DRGs within a medium containing 20 ng/ml triiodothyronine (T3). After co-culture for eight days, td-ciOPLCs differentiated into MBP-positive td-OLs with a typical multi-branching morphology. Notably, many of these td-OLs exhibited extensions along with the surrounding class III β-tubulin (Tuj1)-positive neuron axons, suggesting a myelination (Figure 4). These results validate a myelinogenic ability of ciOPLCs. Thus, our study reveals that the chemical-induced fibroblast-originated ciOPLCs are able to generate functional OLs which myelinate neurons *in vitro*, validating their OPC identity molecularly and functionally.

### Chemical treatment has a general effect on OPC reprogramming

**Figure 5:**
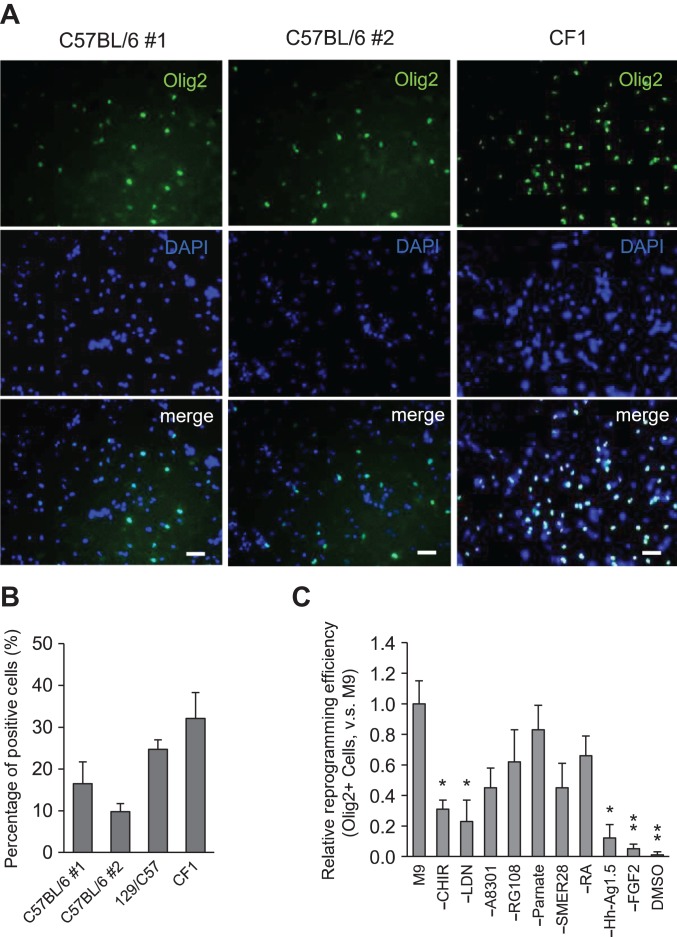
Characterization of the chemical condition. (**A** and **B**) Efficiency of ciOPLC reprogramming for MEFs of C57BL/6 (batch #1 and #2), 129×C57BL/6 (129/C57), and CF1 backgrounds was calculated by counting the percentage of Olig2-positive cells by Day 14 by InCell software. Total cell number was determined by DAPI staining. Data are presented as mean ± SEM. Scale bar, 50 μm. (**C**) Relative reprogramming efficiency of indicated treatments was calculated by InCell software and normalized to that of M9. ‘−’ means removing the indicated component from M9. DMSO served as negative control. Data are presented as mean ± SEM, *n* = 6. *P < 0.01, **P < 0.001.

To further characterize the chemical approach, we applied the reprogramming condition to MEFs of different batch or genetic background. By Day 14, Olig2-positive cells were ~16.5% and 9.8% for two batches of MEFs of C57BL/6 background, 24.72% for MEFs of 129×C57BL/6 background, and 32.1% for MEFs of CF1 background (Figure 5A and B). These results suggest that, in addition to reproducible results with different batches of MEFs, our chemical strategy has a general effect on MEFs with different genetic background.

We next examined the impact of individual components in M9 in OPC induction. We found that removing bFGF, Hh-Ag1.5, and LDN193189 affected the reprogramming efficiency most, as indicated by the percentage of Olig2-positive cells by Day 14 (Figure 5C). This finding is correlated with the roles of FGF, Hedgehog and bone morphogenetic protein pathways play in OPC development in spinal cord, where these signals are required for caudal-ventral patterning (Noll and Miller, 1993; Chambers et al., 2009; Najm et al., 2013).

## Discussion

In this study, we show that transiently treating MEFs with M9, followed by rationally modulating culture signaling recapitulating OPC development, is able to reprogram MEFs into ciOPLCs. Those ciOPLCs showed similar gene expression with primary NSC-derived OPCs, and could be expanded for at least eight passages *in vitro*. Reprogramming efficiency of ciOPLC is comparable with previous reports of TF-mediated conversion of MEFs to OPCs (Najm et al., 2013; Yang et al., 2013). ciOPLCs were bipotent and able to differentiate into OLs and astrocytes *in vitro*, as previously reported (Kondo and Raff, 2000). In addition, ciOPLC-derived OLs myelinated axons *in vitro*, implying their clinical potentials. Through rigorous lineage-tracing assay with established Fsp1-Cre/Rosa26-tdTomato system, we unambiguously excluded the possibility that ciOPLCs were derived from cell types other than fibroblasts. Furthermore, our chemical condition could induce MEFs of different batches/genetic backgrounds into ciOPLCs. These lines of evidence validated our chemical approach to generation of functional OPC from fibroblasts molecularly and functionally. Thus, our study provides an alternative way to PSC-differentiation and TF-mediated reprogramming to produce OPCs from somatic cells. Given the strong clinical interest of OPCs for treating demyelinating diseases, extensive investigations are required in the future to extend the chemical strategy of OPC reprogramming to human fibroblasts or other clinically relevant cell types, and subsequently to optimize and translate chemical approaches into conventional pharmaceutics.

Conceptually, M9 specifically induces a neural program in fibroblasts (lineage-specific cell-activation, CA) that allows the unstable population of intermediate cells to be redirected towards an alternative neural fate by modulating the culture signals (signaling-directed, SD) at the early stage of reprogramming. Therefore, our study provides a non-integrating chemical approach to reprogramming fibroblasts into ciOPLCs, and advances our previously established CASD-reprogramming paradigm into a chemical-based, lineage-specific manner (Supplementary Figure S7).

## Materials and methods

### Cell culture medium

Cell culture was described as the previous report (Zhang et al., 2016). In brief, all fibroblasts were cultured in MEF medium [Dulbecco’s modified Eagle medium (DMEM) supplemented with 10% fetal bovine serum (FBS), 0.1 mM nonessential amino acids (NEAA), and 2 mM Glutamax] in 5% CO_2_ and 20% O_2_ at 37°C. Medium was refreshed every other day. Primary NSCs and NSC line SCR029 were cultured in NSC medium, i.e. basal medium containing 50% Neural basal, 50% DMEM/F12/Glutamax, 1× N2, 1× B27 without vitamin A, 0.075% bovine serum albumin (BSA), and 0.1 mM nonessential amino acids. NSCs were cultured in basal medium with 20 ng/ml bFGF and 20 ng/ml epidermal growth factor (EGF) as previously described in 5% CO_2_ and 20% O_2_ at 37°C. Medium was refreshed daily. OPCs, including primary NSC-derived OPCs and ciOPLCs, were cultured in OPC medium (basal medium supplemented with 20 ng/ml PDGF-AA, 20 ng/ml bFGF, and 200 ng/ml SHH) in 5% CO_2_ and 20% O_2_ at 37°C. Medium was refreshed every other day. OLs derived from both primary NSCs or ciOPLCs were cultured in OL medium [basal medium supplemented with 20 ng/ml T3, 200 ng/ml SHH, 1 nM LDN193189, 5 μM db-cAMP, and 10 ng/ml neurotrophin 3 (NT3)] in 5% CO_2_ and 20% O_2_ at 37°C. Half of the medium was refreshed every other day.

### Chemical conversion of MEFs into ciOPLCs

M9 medium was prepared as described previously (Zhang et al., 2016). M9 was formulated with basal medium supplemented with 3 μM CHIR99021, 100 nM LDN193189, 0.5 μM A83-01, 0.5 μM Hh-Ag1.5, 1 μM retinoic acid, 10 μM SMER28, 10 μM RG108, 2 μM Parnate, and 10 ng/ml bFGF. All components were freshly added into pre-warmed basal medium immediately before formulating the M9 medium. All chemicals used in this study are listed in Supplementary Table S1.

To induce ciOPLC reprogramming, MEFs were seeded at 10000 cells per well into pre-warmed Matrigel-coated 24-well plates in MEF medium. For tdMEF reprogramming, immediately after fluorescence activated cell sorting (FACS), tdMEFs were seeded at 15000 cells per well into pre-warmed Matrigel-coated 24-well plates in MEF medium supplemented with 1 μM Thiazovivin (Tzv) and cultured in 5% CO_2_ and 20% O_2_ at 37°C for 5 h to allow MEFs to attach to the plate. After 5 h, medium was changed to MEF medium without Tzv. After an overnight culture, MEFs were washed twice with 1× Dulbecco’s phosphate-buffered saline (DPBS) and cultured in freshly prepared M9 medium. Cells were cultured in 5% O_2_ and 5% CO_2_ incubator at 37°C. M9 medium was refreshed every other day.

By Day 6, the culture medium was changed to basal medium supplemented with 100 nM LDN193189, 5 μM SB431542, 1 μM retinoic acid, and 200 ng/ml SHH for one day, and then to OPC medium and cultured in 20% O_2_ and 5% CO_2_ at 37°C thereafter. By Day 10, cells were trypsinized, replated into Matrigel-coated plates in OPC medium, and cultured in 20% O_2_ and 5% CO_2_ at 37°C. By around Day 14, cells were trypsinized and subjected to suspension culture in OPC medium in ultralow-attachment 6-well plates. After ~3–5 days in culture, cell spheres were collected, dissociated with trypsin, and transferred into Matrigel-coated 6-well plate in OPC medium as a monolayer culture. Afterwards, the cells were repeatedly cultured in OPC medium on Matrigel-coated plate in 20% O_2_ and 5% CO_2_ at 37°C.

### Immunocytochemistry

Cells were washed once with 1× DPBS and fixed with 4% paraformaldehyde (PFA) at room temperature for 10 min, permeabilized with 0.2% Triton X-100 in 1× DPBS for 10 min, and then blocked with 7.5% BSA for at least 1 h. All primary antibodies were diluted in 7.5% BSA and incubated at 4°C overnight unless indicated. Cells were then washed five times with 1× DPBS for 10 min each at room temperature. Secondary antibodies were purchased from Invitrogen, diluted into 7.5% BSA, and incubated for 1 h at room temperature. Cells were then washed five times with 1× DPBS for 10 min each, and nuclei were stained with DAPI. For O4 staining, cells were incubated live with antibody diluted in 7.5% BSA solution for 20–60 min at 37°C. Cells were then gently rinsed three times with cell medium and fixed with 4% PFA in DPBS. Staining was then completed as detailed above. Antibodies used in this study are listed in Supplementary Table S2.

### OPC differentiation

To differentiate into oligodendrocytes, ciOPLCs were cultured in OL-differentiation medium containing 20 ng/ml T3, 200 ng/ml SHH, 1 nM LDN193189, 5 μM db-cAMP, and 10 ng/ml NT3 for 8–12 days. Half of the medium was refreshed every other day. To differentiate into astrocytes, ciOPLCs were cultured in basal medium containing 10% FBS for 8–12 days. Medium was refreshed every other day.

### In vitro co-culture myelination assay

Primary rat DRG neurons were purchased from Lonza (Catalog number R-DRG-505). Cells were plated on Matrigel-coated coverslips in neuron medium containing 17.5 μg/ml uridine and 7.5 μg/ml 5-fluoro-2-deoxyuridine for five days before seeding ciOPLCs. ciOPLCs were seeded onto pre-established DRGs in the presence of 20 ng/ml T3 for eight days. Immunostaining was performed with antibodies against Tuj1 and MBP.

### Statistics

Each experiment was performed at least three times. The data are presented as the mean ± SEM. *P*-values were calculated using Student’s *t*-test and indicated in the figure legends.

## Supplementary Material

Supplementary Data
