# Supplementary material for: Conversion of mouse fibroblasts into oligodendrocyte progenitor-like cells through a chemical approach
Source: J Mol Cell Biol. 2019 Jan 10;11(6):489–95. doi: 10.1093/jmcb/mjy088 (PMC6604601; doi:10.1093/jmcb/mjy088)
Supplement: Supplementary Data [file mjy088_supplementary.pdf]

Figure S1

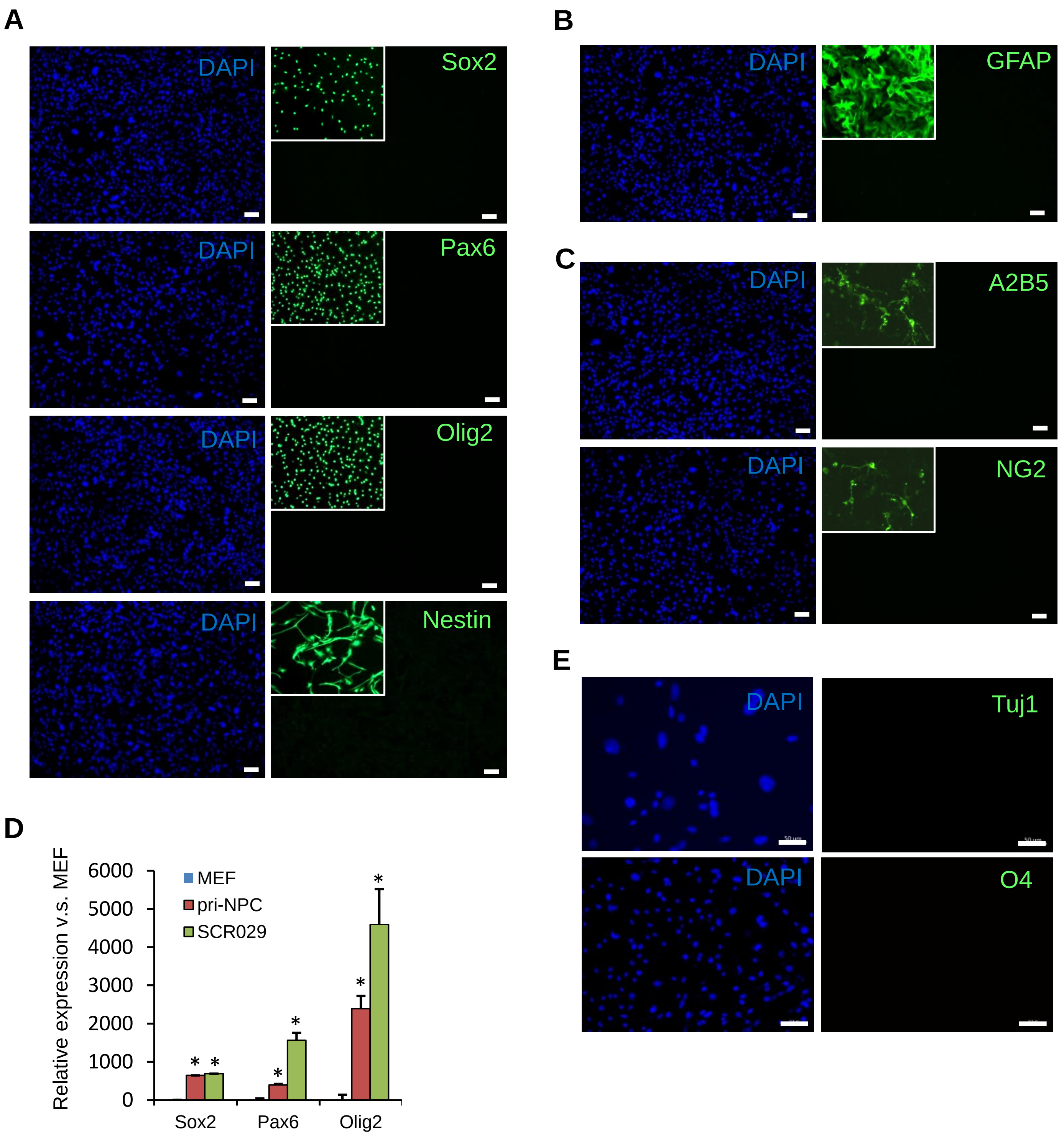

A
B
C
D
E
DAPI
Sox2
Pax6
Olig2
Nestin
GFAP
A2B5
NG2
Tuj1
O4
Relative expression v.s. MEF
MEF
pri-NPC
SCR029
6000
5000
4000
3000
2000
1000
0
Sox2
Pax6
Olig2

Figure S1. Characterization of MEFs Used for Chemical Reprogramming

(A, B and C) Immunostaining analysis showing that the starting MEFs are negative for NSC markers Sox2, Pax6, Olig2, Nestin (A), astroglial marker GFAP (B), and OPC markers A2B5 and NG2 (C). The insets show positive controls for the antibodies. All scale bar, 50 μm.

(D) qRT-PCR analysis showing the expression of indicated neural genes in MEFs. Primary NSCs (pri-NSC) and neural stem cell line SCR029 served as positive controls. Relative expression was normalized to MEFs. Data are represented as mean±SEM. * $p<0.001$.

(E) Immunostaining analysis showing the differentiation potentials for MEFs towards neurons (Tuj1) and oligodendrocytes (O4). Scale bar is 50 μm in the upper panel, and 100 μm in bottom panel.

Figure S2

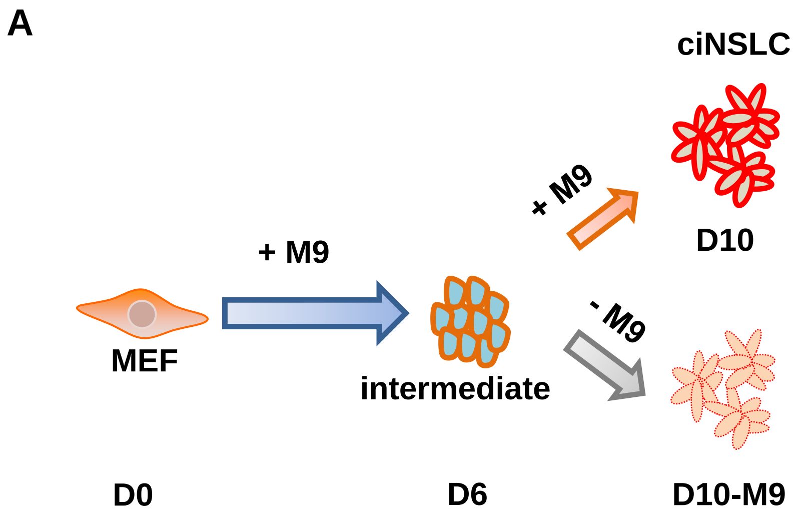
A
ciNSLC
+ M9
D10
+ M9
- M9
MEF
intermediate
D0
D6
D10-M9

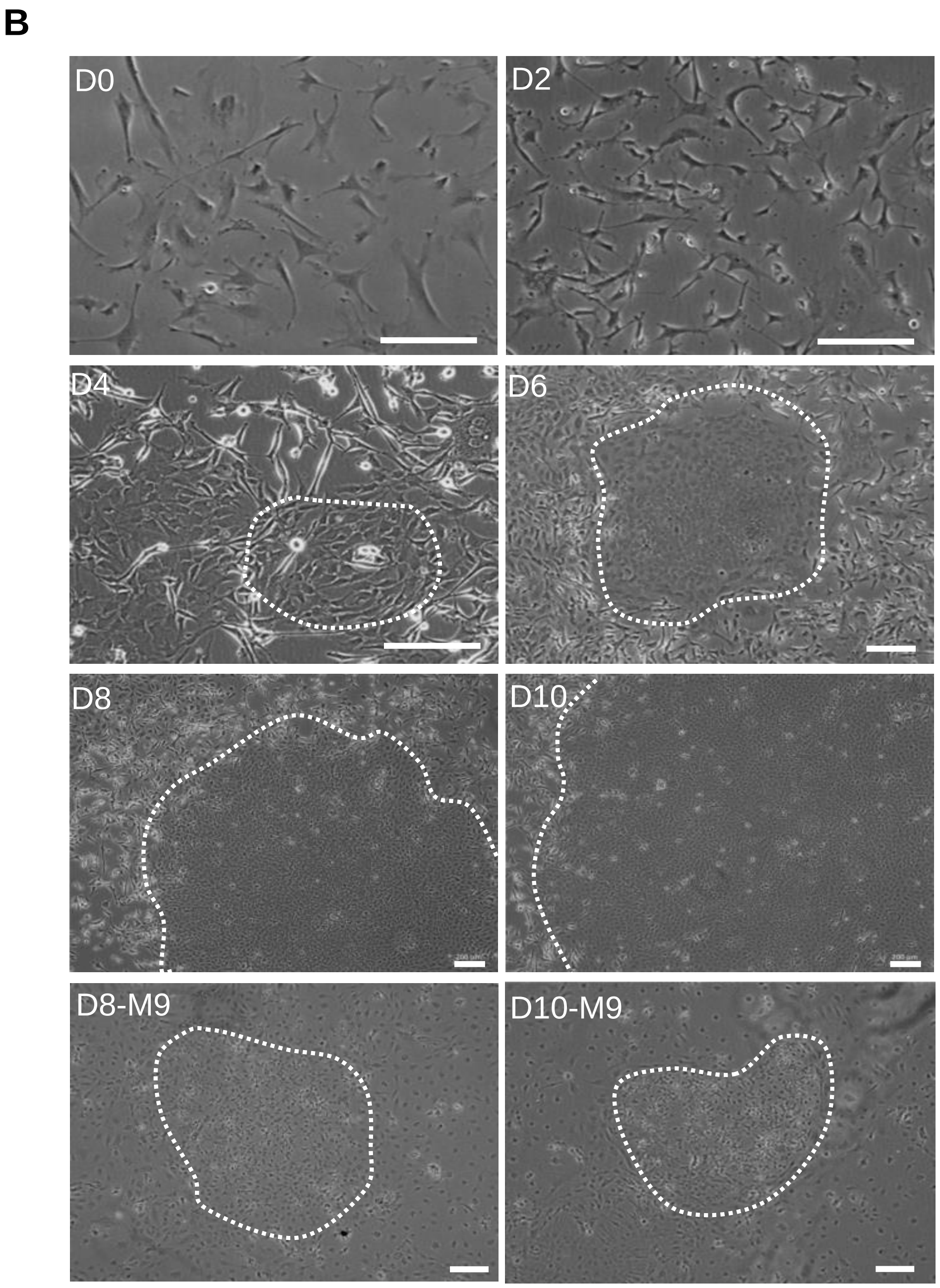
B
D0
D2
D4
D6
D8
D10
D8-M9
D10-M9

Figure S2. M9 Induces the Generation of METed Colonies

(A) A schematic diagram describing the generation, and the differentiation potential, of reprogramming intermediate induced by M9 at the early stage of reprogramming.

(B) Morphology of the METed colonies induced in the presence of M9 at day 2, day 4, day 6, day 8 and day 10 (D2, D4, D6, D8, and D10), or withdraw M9 from day 6 onwards (D8-M9 and D10-M9, M9 was withdraw from D6 and the morphology of the METed colony was recorded by day 8 or day 10, respectively). All scale bar, 200 μm.

Figure S3

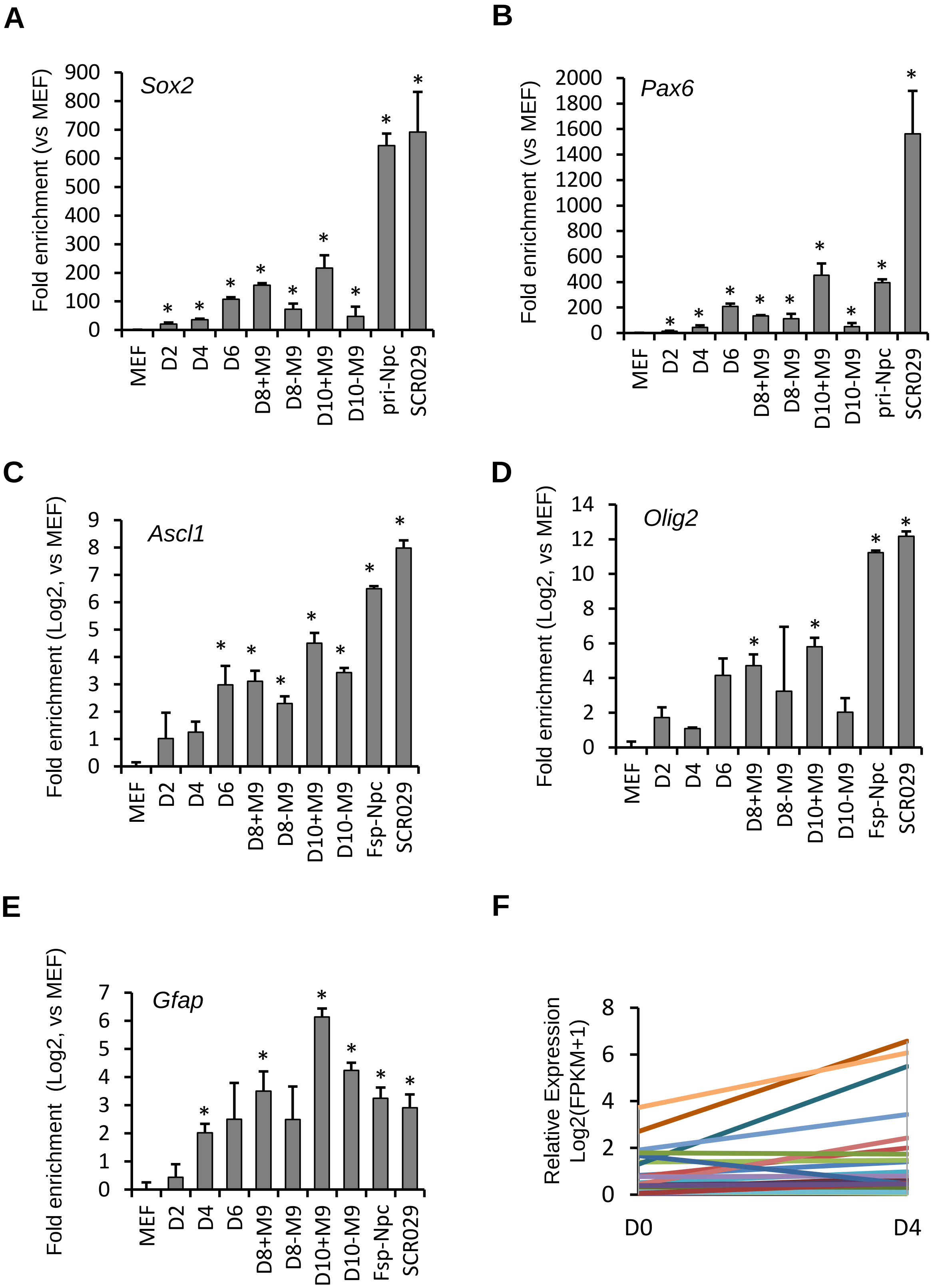
A
Sox2
Fold enrichment (vs MEF)
900
800
700
600
500
400
300
200
100
0
MEF
D2
D4
D6
D8+M9
D8-M9
D10+M9
D10-M9
pri-Npc
SCR029
B
Pax6
Fold enrichment (vs MEF)
2000
1800
1600
1400
1200
1000
800
600
400
200
0
MEF
D2
D4
D6
D8+M9
D8-M9
D10+M9
D10-M9
pri-Npc
SCR029
C
Ascl1
Fold enrichment (Log2, vs MEF)
9
8
7
6
5
4
3
2
1
0
MEF
D2
D4
D6
D8+M9
D8-M9
D10+M9
D10-M9
Fsp-Npc
SCR029
D
Olig2
Fold enrichment (Log2, vs MEF)
14
12
10
8
6
4
2
0
MEF
D2
D4
D6
D8+M9
D8-M9
D10+M9
D10-M9
Fsp-Npc
SCR029
E
Gfap
Fold enrichment (Log2, vs MEF)
7
6
5
4
3
2
1
0
MEF
D2
D4
D6
D8+M9
D8-M9
D10+M9
D10-M9
Fsp-Npc
SCR029
F
Relative Expression
Log2(FPKM+1)
8
6
4
2
0
D0
D4

Figure S3. Gene Expression Analysis of Neural Genes

(A-E) qRT-PCR analysis showing the relative expression of neural genes at indicated time points. Relative expression was normalized to MEFs. Data are represented as mean±SEM, n=3. * $p<0.001$.

(F) RNA-seq results (day 4 and day 0) showing the relative expression of 22 neural genes.

Figure S4

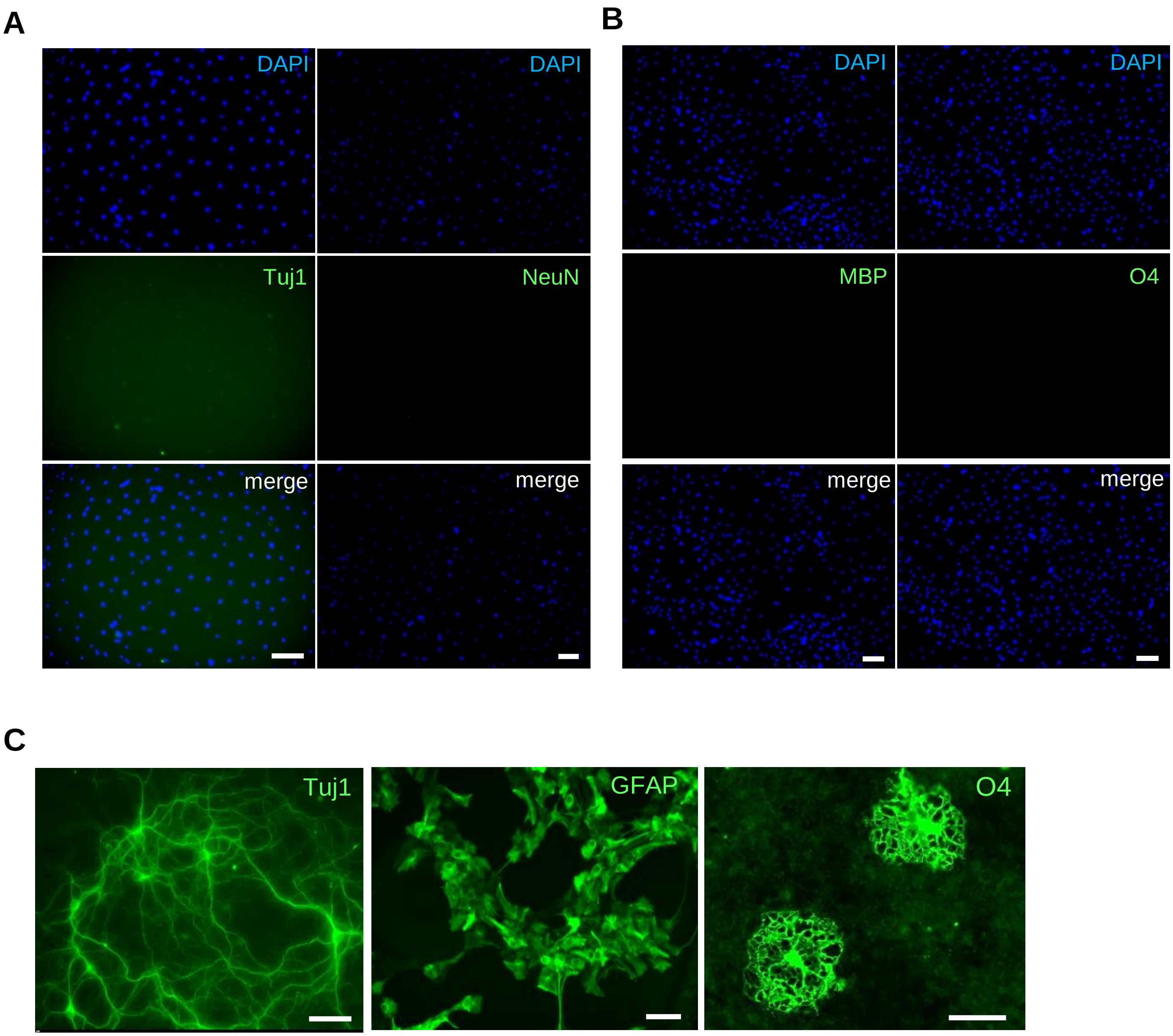
A
DAPI
DAPI
Tuj1
NeuN
merge
merge
B
DAPI
DAPI
MBP
O4
merge
merge
C
Tuj1
GFAP
O4

Figure S4. Differentiation Potentials of M9-Induced Intermediate Cells

Immunostaining analysis showing the M9-induced intermediate cells at day 6 are not able to generate neurons, indicated by Tuj1 and NeuN staining (A), or OLs, indicated by MBP and O4 staining (B). All scale bar, 100 μm.

(C) Immunostaining analysis showing the differentiation potentials for primary neural stem cells towards neurons (Tuj1) astrocytes (GFAP), and oligodendrocytes (O4). All scale bar, 50 μm.

Figure S5

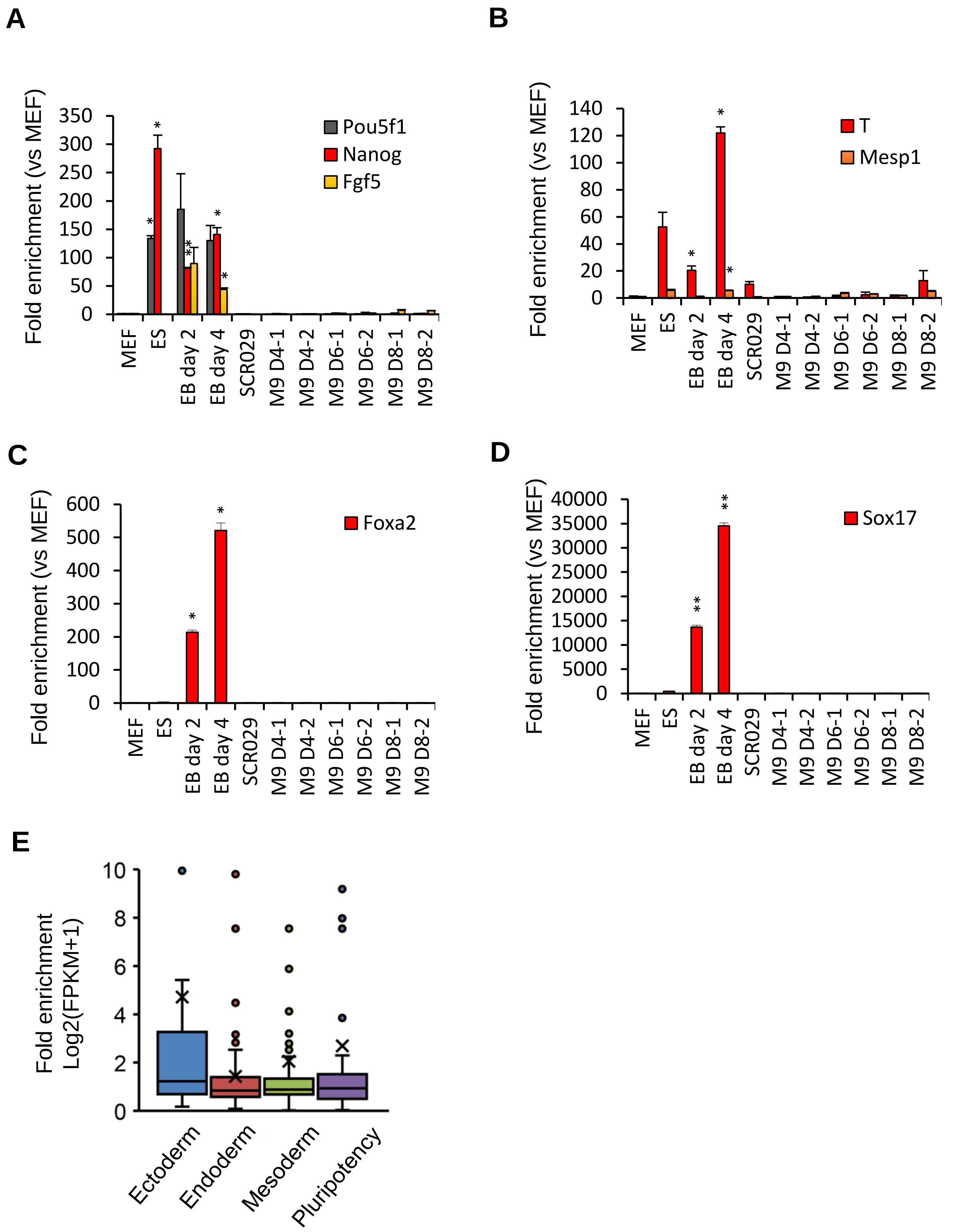

A
Fold enrichment (vs MEF)
Pou5f1
Nanog
Fgf5
MEF
ES
EB day 2
EB day 4
SCR029
M9 D4-1
M9 D4-2
M9 D6-1
M9 D6-2
M9 D8-1
M9 D8-2
B
Fold enrichment (vs MEF)
T
Mesp1
C
Fold enrichment (vs MEF)
Foxa2
D
Fold enrichment (vs MEF)
Sox17
E
Fold enrichment Log2(FPKM+1)
Ectoderm
Endoderm
Mesoderm
Pluripotency

Figure S5. Gene Expression Analysis of Lineage-Specific Genes

(A-D) qRT-PCR analysis showing the expression of genes for pluripotency (A), mesoderm (B) and endoderm (C, D), respectively, at indicated time points. EB, embryoid body. Relative expression was normalized to MEFs. Data are represented as mean±SEM. * $p<0.01$, ** $p<0.001$.

(E) Box plots showing the fold enrichment of the expression of genes for ectoderm, endoderm, mesoderm and pluripotency (day 4 v.s. day 0).

Figure S6

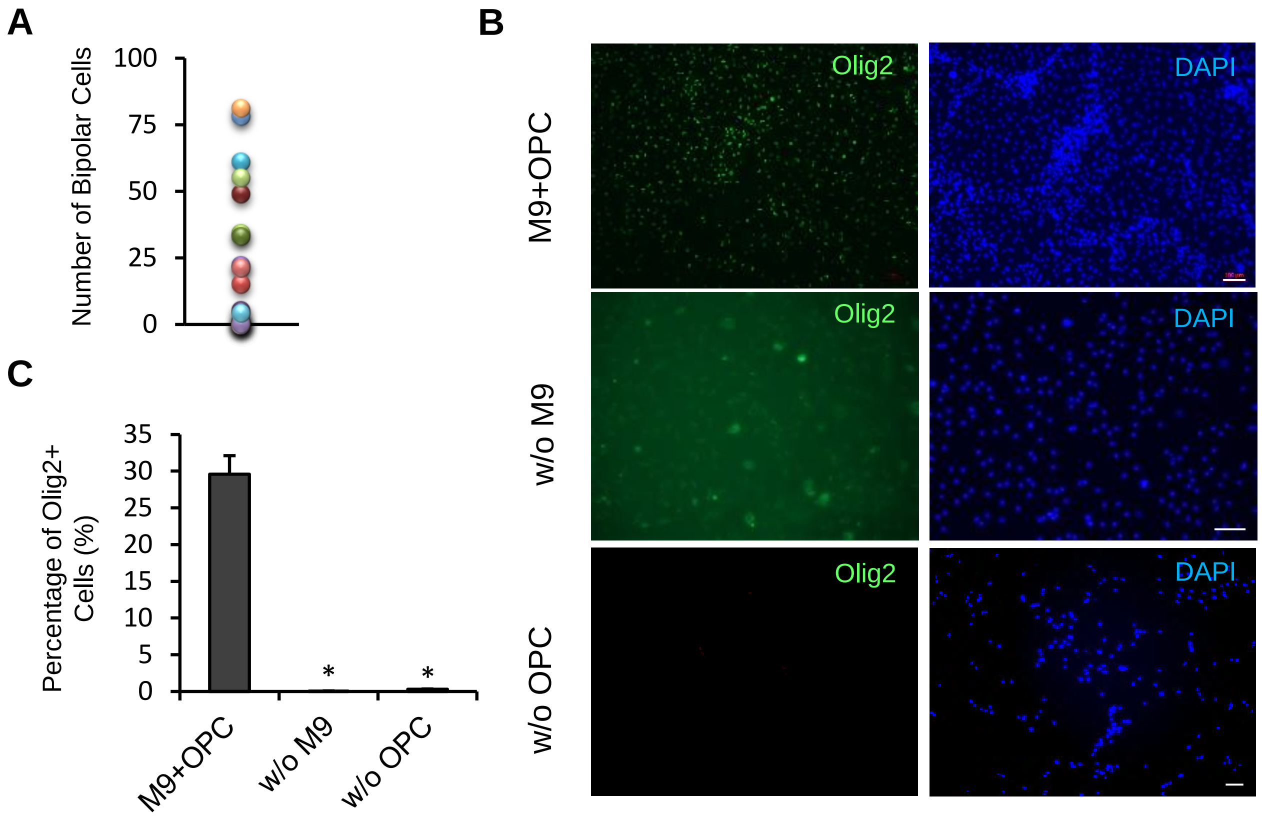

A
Number of Bipolar Cells
100
75
50
25
0
B
M9+OPC
w/o M9
w/o OPC
Olig2
DAPI
C
Percentage of Olig2+ Cells (%)
35
30
25
20
15
10
5
0
*
*
M9+OPC
w/o M9
w/o OPC

Figure S6. Characterization of Reprogramming Conditions

(A) Dot plot showing the quantification of the numbers of bipolar cells by counting 18 randomly picked 10× visual fields from three independent experiments (6 fields for each experiment).

(B and C) Immunostaining showing the Olig2-positive cells (B) and the quantification (C) by day 14 under the conditions with M9 and OPC treatment (upper panel), without M9 for the first 6 days (middle panel), or without OPC specification after day 6 (bottom panel). All scale bar, 100 μm. * $p<0.01$.

Figure S7

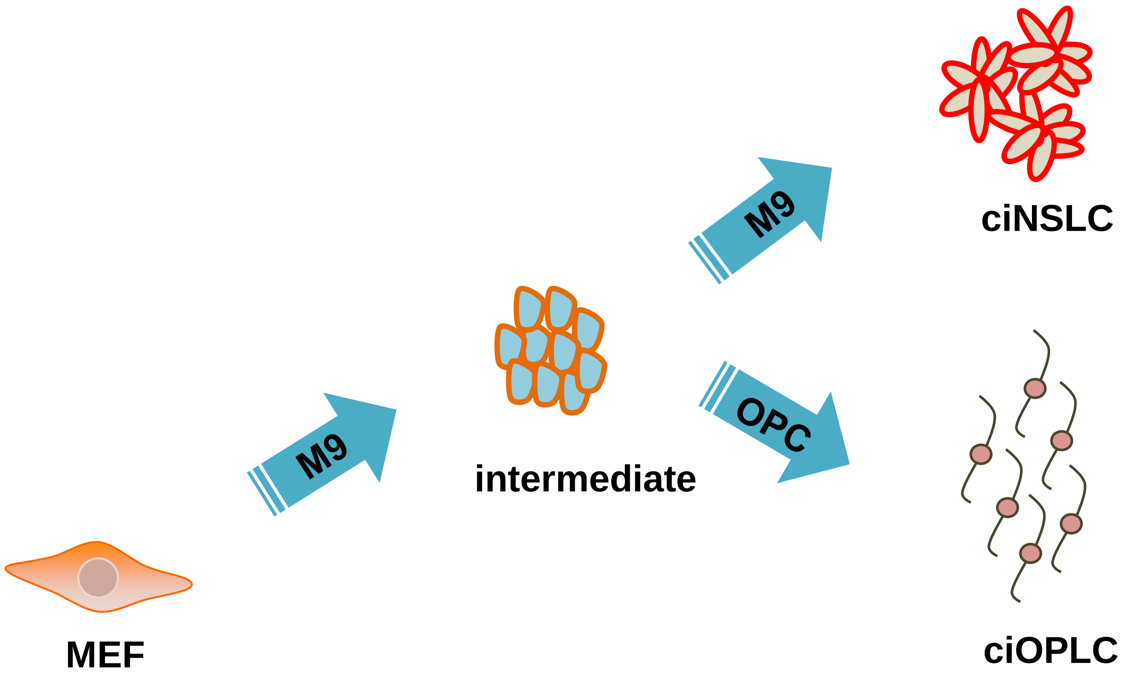

Figure S7. Working Model of ciOPLC Reprogramming

Similar with previously described TFs-mediated Cell Activation and Signaling Directed reprogramming paradigm, chemical condition could activate fibroblasts into an epigenetically active state (the METed colony), which could be re-directed to alternative OPC fate in appropriate culture conditions.

SUPPLEMENTARY TABLES

Supplementary Table S1. Chemicals included in M9 cocktail

Chemical	Function	Structure	Working Con.	M.W.
CHIR99021	Highly potent and selective GSK-3 inhibitor	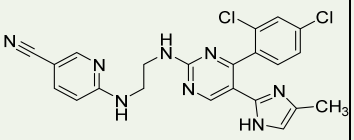	3 μM	465.34
LDN193189	Cell permeable BMP type I receptors ALK2/ALK3 inhibitor	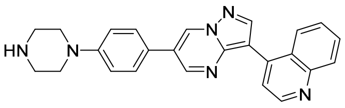	100 nM	406.48
A83-01	Potent inhibitor of TGF-β type I receptor ALK5 kinase, type I Activin/Nodal receptor ALK4 and type I nodal receptor ALK7.	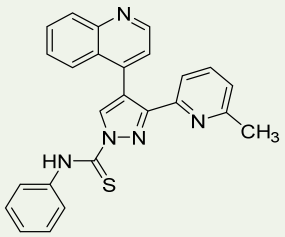	0.5 μM	421.52
Parnate	Irreversible inhibitor of lysine-specific demethylase 1 and monoamine oxidase	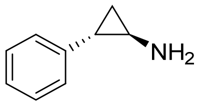	2 μM	169.65
RG108	Non-nucleoside DNA methyltransferase inhibitor	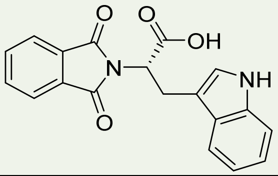	10 μM	334.3
Retinoic Acid	Endogenous agonist for retinoic acid receptors and retinoid X receptor	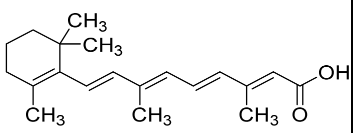	1 μM	300.44

SMER28	Positive regulator of autophagy	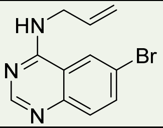	10 μM	264.12
Hg-Ag 1.5	Potent Hedgehog pathway Smo agonist	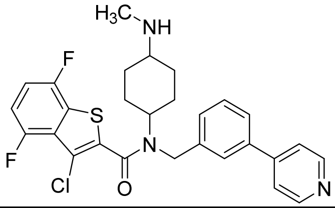	0.5 μM	526.04

Supplemental Table S2. Primary Antibodies Used in This Study

Antigen	Antibody Type	Company	Cat No
Tuj1	mouse mAb	Covance	MMS-435P
Nestin	mouse IgG2a	R&D	MAB2736
Pax6	Rabbit	Covance	PRB-278P
GFAP	rabbit	Dako	z0334
Olig2	rabbit	Millipore	AB9610
O4	mouse mAb	R&D	MAB1326
MBP	mouse	Convance	SMI99P
MAG	mouse	Millipore	MAB1567
MOG	mouse	Millipore	MAB5680
NG2	rabbit	Millipore	AB5320
A2B5	mouse	R&D	MAB1416
PDGFRa	mouse	eBioscience	17-1401-81
Nkx2.2	mouse	DSHB	74.5A5
Isotype control Ab	mouse	eBioscience	17-4321-81

Supplemental Table S3. Quantitative PCR Primers Used in this Study

Gapdh	forward primer	catggccttccgtgttccta
	reverse primer	gcctgcttcaccaccttctt
Sox2	forward primer	gaacgccttcatggtatggt
	reverse primer	ttgctgatctccgagttgtg
Gfap	forward primer	cggagacgcatcacctctg
	reverse primer	agggagtggaggagtcattcg
Olig2	forward primer	ggcggtggcttcaagtcatc
	reverse primer	tagtttcgcgccagcagcag
Ascl1	forward primer	agggatcctacgaccctctta
	reverse primer	accagttggtaaagtccagcag
Pax6	forward primer	agggggagagaacaccaact
	reverse primer	catttggcccttcgattaga
Nkx2.2	forward primer	gcagcgacaacccctaca
	reverse primer	acttggagctcgagtcttgg
Mbp	forward primer	taccctggctaaagcagagc
	reverse primer	gggagccgtagtgggtagtt
Plp1	forward primer	ctgccagtctattgccttcc
	reverse primer	agcattccatgggagaacac
Sox6	forward primer	caaaggacgaaaggaggaaa
	reverse primer	ggatttccagcgagatccta
Pou5f1	forward primer	ctgagggccaggcaggagcacgag
	reverse primer	ctgtagggagggcttcgggcactt
Nanog	forward primer	aag cag aag atg cgg act gt
	reverse primer	atc tgc tgg agg ctg agg ta
Fgf5	forward primer	ccttgcgacccaggagctta
	reverse primer	ccgtctgtggtttctgttgagg
Foxa2	forward primer	aagggaaatgagaggctgagtgga
	reverse primer	atgacagatcactgtggcccatct
T	forward primer	ttgaactttcctccatgtgctga
	reverse primer	tcccaagagcctgccacttt
Mesp1	forward primer	acccatcgttccagtacgc
	reverse primer	agcatgtcgctgctgaaga
Sox17	forward primer	tgccctttgtgtataagcccgaga
	reverse primer	gggtagttgcaatagtagaccgct

SUPPLEMENTAL INFORMATION

Materials and Methods

Mouse Embryonic Fibroblasts Preparations

Procedures involving mice were approved by the Animal Care and Use Committee at the Shanghai Jiao Tong University School of Medicine. Mouse embryonic fibroblasts (MEFs) were prepared as reported (Zhang et al., 2016). In brief, E13.5 mouse embryos with the desired genotype were collected and their neural tissues (including head, spinal cord, and tail) and internal organs (including reproductive organs) were carefully removed. The remaining tissues were sliced into small pieces, trypsinized, plated in MEF medium, and cultured in 5% CO_2 and 20% O_2 at 37°C. All fibroblasts were expanded for two passages before being used for experiments. To prepare tdMEFs, the resulting fibroblasts were sorted for tdTomato$^+$/p75$^-$ cells by fluorescence-activated cell sorting (FACS).

RNA Preparation and RT-PCR

Total RNA was extracted using the RNeasy Plus mini kit (Qiagen). Reverse transcription and PCR were performed as described. In brief, 1 μg total RNA was used for reverse transcription reactions with the iScript cDNA synthesis kit (Bio-Rad), and the resulting cDNA was diluted five times in H_2O for PCR. For semi-quantitative PCR, 1 μl of 1/5-diluted cDNA was used as a template for the PCR program: 95°C for 5 min, 35 cycles of 95°C for 30 s, 60°C for 30 s, and 72°C for 30 s, and then 72°C for 10 min. Quantitative PCR was performed following the protocol of FAST SYBR Green Master Mix (Applied Biosystems). All PCR was performed in triplicate, and the expression of

individual genes was normalized to that of *Gapdh*. The primer sequences are listed in Supplemental Table S3.

FACS Cytometry

Cells were washed twice with 1×DPBS and detached with accutase treatment at 37°C for 5 min. After harvesting, cells were passed through a 70-μm filter and washed twice with and resuspended into pre-cooled FACS buffer (1×DPBS, 1.5% FBS, 0.5% BSA). Cells were incubated with specific antibodies or isotype control with the suggested concentrations on ice for 45 min, followed by six washes with FACS buffer for sorting. Cells were then resuspended into FACS buffer and sorted with a BD FACSAris II. Antibodies used in this study are listed in Supplemental Table S2.
